# Imaging of Gα_q_ Proteins in Mouse and Human Organs and Tissues

**DOI:** 10.3390/pharmaceutics15010057

**Published:** 2022-12-24

**Authors:** Jan H. Voss, Haneen Al-Hroub, Robin Gedschold, Jennifer M. Dietrich, Evelyn Gaffal, Marieta Toma, Stefan Kehraus, Gabriele M. König, Peter Brust, Bernd K. Fleischmann, Daniela Wenzel, Winnie Deuther-Conrad, Christa E. Müller

**Affiliations:** 1 PharmaCenter Bonn, Pharmaceutical Institute, Pharmaceutical & Medicinal Chemistry, University of Bonn, D-53121 Bonn, Germany; 2 Institute of Physiology I, Life & Brain Center, Medical Faculty, University of Bonn, D-53127 Bonn, Germany; 3 Department of Dermatology, University Hospital Magdeburg, D-39120 Magdeburg, Germany; 4 Institute of Pathology, University Hospital Bonn (UKB), Medical Faculty, University of Bonn, D-53127 Bonn, Germany; 5 Institute for Pharmaceutical Biology, University of Bonn, Nussallee 6, D-53115 Bonn, Germany; 6 Department of Neuroradiopharmaceuticals, Institute of Radiopharmaceutical Cancer Research, Helmholtz-Zentrum Dresden-Rossendorf, D-03418 Leipzig, Germany; 7 Department of Systems Physiology, Institute of Physiology, Medical Faculty, Ruhr University of Bochum, D-44801 Bochum, Germany

**Keywords:** asthma, autoradiography, G protein-coupled receptors, Gα_q_ protein, FR900359, lung, melanoma, mouse brain, radioligand, YM-254890

## Abstract

G protein-coupled receptors (GPCRs) transfer extracellular signals across cell membranes by activating intracellular heterotrimeric G proteins. Several studies suggested G proteins as novel drug targets for the treatment of complex diseases, e.g., asthma and cancer. Recently, we developed specific radiotracers, [³H]PSB-15900-FR and [³H]PSB-16254-YM, for the Gα_q_ family of G proteins by tritiation of the macrocyclic natural products FR900359 (FR) and YM-254890 (YM). In the present study, we utilized these potent radioligands to perform autoradiography studies in tissues of healthy mice, mouse models of disease, and human tissues. Specific binding was high, while non-specific binding was extraordinarily low, giving nearly identical results for both radioligands. High expression levels of Gα_q_ proteins were detected in healthy mouse organs showing the following rank order of potency: kidney > liver > brain > pancreas > lung > spleen, while expression in the heart was low. Organ sub-structures, e.g., of mouse brain and lung, were clearly distinguishable. Whereas an acute asthma model in mice did not result in altered Gα_q_ protein expressions as compared to control animals, a cutaneous melanoma model displayed significantly increased expression in comparison to healthy skin. These results suggest the future development of Gα_q_-protein-binding radio-tracers as novel diagnostics.

## 1. Introduction

Heterotrimeric guanine nucleotide-binding proteins (“G proteins”) play a key role in cellular signaling by transmitting information from activated G protein-coupled receptors (GPCRs) to intracellular effectors. G proteins consist of three subunits, α, β, and γ [1]. The Gα protein subunit is the main interaction partner for GPCRs and their effector proteins. There are four families of Gα proteins, Gα_s_, Gα_i/o_, Gα_q/11_, and Gα_12/13_ proteins, each of which is associated with distinct intracellular signaling pathways [2,3]. The Gα_q/11_ (“Gα_q_”) protein family comprises four members (Gα_q_, Gα_11_, Gα_14_, and Gα_15_) that link GPCRs to phospholipase C-β (PLC-β) activation. PLC-β hydrolyzes phosphatidylinositol-4,5-bisphosphate to inositol trisphosphate (IP_3_) and diacylglycerol (DAG), leading to calcium mobilization and protein kinase C activation [4,5,6].

Gα_q_ and Gα_11_ proteins are ubiquitously expressed, sharing a sequence identity of 90% [7,8], and they are regarded as functionally equivalent with respect to receptor interaction [9]. The Gα_14_ protein shares 80% sequence identity with the Gα_q_ protein. Its mRNA is widely transcribed, with the highest transcription levels in endocrine tissues [10], and it is activated by the same GPCRs as the Gα_q_ protein [9,11]. The Gα_15_ protein shows the largest evolutionary distance to the Gα_q_ protein, sharing only 54% of its sequence identity. Its expression is limited to the hematopoietic system, which suggests a specific role for the Gα_15_ protein in immune function [12,13]. The Gα_15_ protein is activated by a wider range of GPCRs and is therefore considered to be a promiscuous Gα protein [9,11,14].

The Gα_q_ family of proteins are important signal transducers in mammalian cells [15] that can be activated by approximately 45% of therapeutically relevant human GPCRs [9]. Hyperactivation of Gα_q_-mediated signaling pathways plays a role in several diseases, such as uveal melanoma [16,17], asthma bronchiale [18], pulmonary hypertension [19], and cardiac hypertrophy [20]. The macrocyclic natural products YM-254890 (YM) and FR900359 (FR) are potent and selective inhibitors of Gα_q_-family proteins, namely of the Gα_q_, Gα_11_, and Gα_14_ protein subunits. In their hydrogenated, [³H]-labeled form, they were previously shown to bind to mouse and human Gα_q/11/14_ proteins with high affinity in the low nanomolar range, low non-specific binding, and, in the case of FR, a long residence time [21]. FR was found to display a ~1000-fold lower potency at the Gα_15_ protein, while YM did not appear to inhibit the Gα_15_ protein at all [22]. Both compounds showed no off-target effects, even at high inhibitor concentrations of up to 100 µM [23]. Therefore, derivatives of YM and FR may have ideal properties for the development of diagnostic radiotracers to detect pathologies that show altered Gα_q_ protein expression relative to healthy tissues.

In the present study, we investigated the suitability of radiolabeled macrocyclic Gα_q_ protein inhibitors as in vitro diagnostics (i) to evaluate Gα_q_ protein expression on the protein level, (ii) to image their distribution in different organs and tissues, and (iii) to identify disease conditions that may show up- or downregulation of Gα_q_ protein expression. To this end, we performed autoradiography experiments using the selective, high-affinity FR- and YM-derived radiotracers, [³H]PSB-15900-FR and [³H]PSB-16254-YM, labeling the closely related Gα_q_ protein family members Gα_q_, Gα_11_, and Gα_14_. Gα_q_ protein expression was analyzed in tissues and organs from healthy mice, an asthma mouse model, mouse melanoma, and human tissues.

## 2. Materials and Methods

### 2.1. Organs from CD1 Mice

Female CD1 wild-type mice were housed with ad libitum chow and water supply under a normal circadian rhythm. At the age of 10 weeks, mice were killed by cervical dislocation. Respective organs were harvested from three healthy animals, frozen in isopentane, and stored at −20 °C until use. Animal experiments were carried out in accordance with the guidelines of the German law of protection of animal life with approval by the local government authorities (Landesdirektion Sachsen, No. DD24.1–5131/446/19; TVV 18/18).

### 2.2. Acute Asthma Model and Preparation of Fixated Lung Tissue

Female BALB/c mice were housed with ad libitum chow and water supply under a normal circadian rhythm until the age of 10 weeks. On day 0 and day 14, mice were sensibilized by intraperitoneal injection of 20 µg ovalbumin adsorbed to Imject Alum (2 mg/mL). On days 21, 22, and 23, the mice were challenged by a nebulized 1% ovalbumin solution. The mice were sacrificed on day 24. Control mice were untreated.

To fixate lung tissue, the trachea was isolated and punctured and the lung was filled with modified OCT compound (100 mL Hanks’ balanced salt solution (HBSS) with 10 g polyvinyl alcohol, heated in a microwave; when cooled down to room temperature, 8 mL polypropylene glycol 2000 and 100 mg sodium azide were added) and sealed with a thread. Heart and lung were removed from the mice, washed with HBSS, and separated afterwards. Organs were snap-frozen in liquid nitrogen and stored at −80 °C until cryosectioning. Animal experiments were carried out in accordance with the guidelines of the German law of protection of animal life with approval by the local government authorities (Landesamt für Natur, Umwelt and Verbraucherschutz Nordrhein-Westfalen, NRW, Germany, Az:81-02.04.2018.A297)

### 2.3. Mouse Melanoma

B16 (F1) is a spontaneous murine melanoma. The B16-F1 cell line (Gα_q/11_ wt) was maintained in RPMI supplemented with 10% heat-inactivated FCS, 2 mmol/L l-glutamine, 50 μmol/L 2-mercaptoethanol, 100 IU/mL penicillin, and 100 μg/mL streptomycin. Engraftment of B16 melanoma in the skin was conducted by intracutaneous injection of 1 × 10^5^ B16 melanoma cells. Mice with tumors >100 mm^2^ were sacrificed. Tumors as well as healthy mouse skin were harvested and snap-frozen for further analyses. Animal experiments were carried out in accordance with the guidelines of the German law with approval by the local government authorities (Landesverwaltungsamt Halle, Sachsen-Anhalt, Germany, Az. 42502-2-1556, 20 June 2019).

### 2.4. Human Cancer Samples

Fresh material from patients who underwent surgery for malignant tumors was received in the Institute of Pathology, University Hospital Bonn. After macroscopically inspection of the specimens, representative tumor tissue (5–10 mm^3^), as well as non-neoplastic tissue, were sampled and snap-frozen in liquid nitrogen. The samples were anonymized and transferred to the biobank storage at −80 °C. All patients were given informed consent for biobanking and the study was approved by the ethics committee of the University Hospital Bonn (EK285/21; 21 July 2021). The study was conducted in accordance with the Declaration of Helsinki. Human squamous cell carcinoma specimens were obtained from surgically removed tumors and snap-frozen. Healthy skin was taken from the same patient All specimen were taken according to local ethical guidelines. Patients had given informed consent, and the study was approved by the ethics committee of the Otto-von-Guericke University of Magdeburg (AZ 162/20).

### 2.5. Cryosectioning

Frozen organs were adjusted to the temperature of the cryostat (CryoStar NX70, Thermo Fisher Diagnostics GmbH, Henningsdorf, Germany) and mounted on a carrier plate with Tissue-Tek^®^ O.C.T.^TM^ compound (Sakura Finetek, Alphen an de Rijn, The Netherlands). The organs were trimmed until tissue slices spanned the entire cross-section of the respective organ. Slices with a thickness of 10 µm (lung: 16 µm) were cut and mounted onto room-tempered microscopic glass slides (2–4 slices per slide). Tissue slices were dried for 60 min and stored at −20 °C.

### 2.6. Histological Staining of Samples

Slices of each harvested organ were stained by Nissl-staining and Hematoxylin-Eosin (HE)-staining. For Nissl-staining, slices were thawed, dried, and the tissue slices were covered in Nissl staining solution (1.5% cresyl violet in aqueous ammonium acetate buffer, pH 4.6) for 5 min. The slides were subsequently rinsed and flushed with deionized water, differentiated in 70% and 100% ethanol, rinsed, and dried. HE staining was performed with a HE-staining kit (BIOZOL, Eching, Germany). Dried tissue slices were covered in Hematoxylin staining solution for 5 min, rinsed with deionized water briefly, and incubated in warm tap water for bluing for 3–5 min. Slides were dipped in 100% ethanol for 10 s and subsequently incubated with Eosin Y solution for 3 min. After rinsing the slides with 100% ethanol, they were dehydrated with 100% ethanol for 3 min and dried. When dry, the coverslip was mounted onto the tissue section with NeoMount (Merck Millipore, Burlington, MA, USA) and fixed with a weight until the mounting medium solidified. Each slide was scanned in high resolution.

### 2.7. Autoradiography

Radioligand ([³H]PSB-15900-FR, molar activity 1036 GBq mmol^−1^ (28 Ci mmol^−1^), [³H]PSB-16254-YM, molar activity 1147 GBq mmol^−1^ (31 Ci mmol^−1^)) solutions were prepared in autoradiography buffer (25 mM Tris, 120 mM NaCl, 5 mM KCl, 2 mM CaCl_2_, 1 mM MgCl_2_; pH_RT_ 7.4) at a final concentration of 10 nM. For the determination of total binding, 1% DMSO was added to the solution. Non-specific binding was recorded in the presence of 5 µM FR900359 (final DMSO concentration: 1%). Slides mounted with cryosectioned tissue slices were thawed, dried, and pre-treated with autoradiography buffer for 15 min. The excess buffer was removed from the slides. When dry, the slides were covered in radioligand solution for 60 min. Subsequently, the radioligand solution was decanted and the slides were incubated in ice-cold 50 mM Tris, pH 7.4 twice for 2 min per step. Buffer salts were removed by dipping all slides in ice-cold deionized water for 10 s. Each organ was analyzed in triplicate (organs were harvested from 3 individual mice; 2 individual mice for experiments with asthmatic mice) and for each harvested organ, 2 slides of total binding and 2 slides of non-specific binding were imaged. Afterwards, slides were mounted onto an imaging plate (BAS TR2025, FUJIFILM, Minato, Japan) and incubated for 3 weeks before scanning the plates with a phosphoimager (CR35 NDT, Dürr NDT GmbH & Co. KG, Bietigheim-Bissingen, Germany).

### 2.8. Densitometric Analysis

Images of the scanned plates were analyzed with AIDA (v 4.27). Each pixel in the obtained image had a size of 12.61 µm × 12.61 µm. Regions of interest (ROI) were manually defined within each tissue section. Generally, the average gray-scale intensity of each ROI was determined; in lung and brain sections, the average gray-scale intensity of discernible substructures were determined as well. ROIs were defined from the autoradiograms alone. A calibration curve of tritium standards (ART0123B/C; American Radiolabeled Chemicals, St. Louis, MO, USA) with a known activity (in Bq/mg of tissue equivalent) was used to transform gray-scale intensity of the image into activity per mg of tissue (in nCi per mg of tissue). Taking the molar activity of the radioligand into account, these values are transformed into pmol radioligand bound per mg tissue. Subsequently, the non-specific binding was subtracted from the total binding of the samples to obtain the specific radioligand binding per mg of tissue. Considering the K_D_ values of each radioligand (*p*K_D_ [³H]PSB-15900-FR, 7.92; *p*K_D_ [³H]PSB-16254-YM, 7.80, determined by saturation binding to HEK cell membranes expressing the Gα_q_ protein at 37 °C [24]), and the radioligand concentration, B_max_ values (in pmol Gα_q/11_ proteins per mg of tissue) were calculated for each region of interest by inserting the respective parameters into a one-site saturation binding equation.

Numeric values were further analyzed in GraphPad PRISM 8.4.0 (GraphPad, San Diego, CA, USA). When evaluating the statistical significance of a difference between two means, an unpaired *t*-test was used (if necessary corrected for multiple comparisons). Significance analysis among three or more mean values was computed with a one-way analysis of variance (ANOVA). Dunnett’s post-hoc test was employed to obtain significance levels of each mean compared to a reference mean value. Statistical significance was presented as follows: *p* < 0.05, *; *p* < 0.01, **, *p* < 0.001, ***.

## 3. Results

### 3.1. Autoradiography of Gα_q_ Proteins in Healthy Mouse Brain

**Figure 1:**
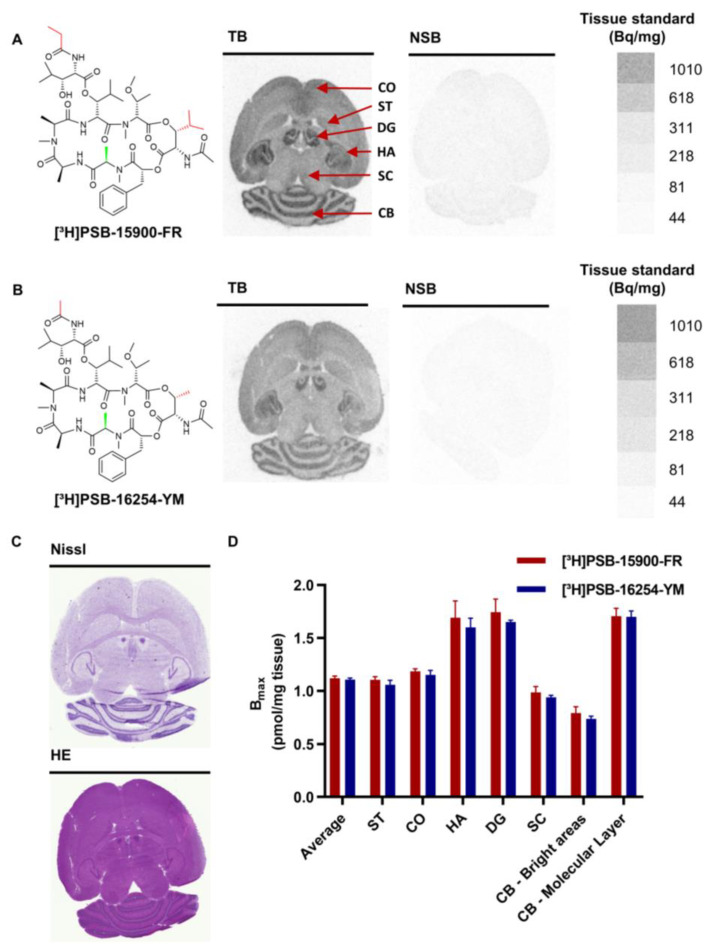
Chemical structures of the employed radioligands and representative autoradiography images of mouse brain sections, incubated with (**A**) [³H]PSB-15900-FR and (**B**) [³H]PSB-16254-YM. Images display total radioligand binding (TB) and non-specific binding (NSB; determined in presence of 5 µM FR). Organ slices were incubated with 10 nM of the indicated radioligand for 1 h at room temperature. Tissue equivalent standards for each radioligand are displayed on the righthand side. Red arrows point to the indicated brain regions: ST, striatum; CO, cortex; HA, hippocampus; DG, dentate gyrus; SC, superior colliculus; CB, cerebellum. (**C**) Brain sections were stained with cresyl violet (Nissl) and hematoxylin-eosin (HE). (**D**) B_max_ values obtained by densitometric analysis of radioligand binding.

The expression of Gα_q_ proteins was determined by autoradiography in different tissues including the brain, lung, heart, liver, kidney, pancreas, and spleen from healthy female CD1 mice. Both radioligands, [³H]PSB-15900-FR and [³H]PSB-16254-YM, were compared by incubating brain sections with each radioligand (Figure 1A,B). High specific binding and minimal non-specific binding, determined in the presence of unlabeled FR900359 (5 µM), were observed for either radioligand. Moreover, they showed identical binding patterns in different brain regions. Brain sections were additionally stained with cresyl violet (Nissl staining; blue staining of basophilic molecules, e.g., RNA and DNA, thereby labeling neuronal cell bodies) and hematoxylin-eosin (blue staining of basophilic structures and red staining of eosinophilic structures) (Figure 1C). In the hippocampus and cerebellum, radiotracer binding correlated inversely with Nissl-staining, i.e., cell body-rich regions displayed low radioligand binding, while regions mostly composed of nerve fibers showed high radiotracer binding.

The expression of Gα_q_ proteins was calculated by densitometric analysis (Figure 1D, Table 1, see Methods section for details of the calculation), resulting in an average expression in the brain of 1.12 pmol per mg tissue (calculated for binding of [³H]PSB-15900-FR). Higher radioligand binding was found in the dentate gyrus (DG, 1.74 pmol/mg), hippocampal areas (HA, 1.69 pmol/mg), and the molecular layer of the cerebellum (CB). Cortex (CO, 1.18 pmol/mg) and striatum (ST, 1.12 pmol/mg) displayed intermediate Gα_q_ protein expression levels, while superior colliculus (SC) and the bright areas of the cerebellum (composed of the Purkinje cell layer, granular cell layer, and white matter) showed lower Gα_q_ protein expression (0.99, and 0.79 pmol/mg, respectively).

Both radioligands can be considered suitable for autoradiography studies due to their high affinity (pK_D_ [³H]PSB-15900-FR = 8.19, pK_D_ [³H]PSB-16254-YM = 7.80 [24]), very low nonspecific binding, and their ability to discriminate between regions with lower and higher Gα_q_ protein expression. Because of similar results for both radioligands in initial experiments, we decided to perform further autoradiography studies with [³H]PSB-15900-FR due to its longer residence time at the Gα_q_ proteins [21,24].

### 3.2. Various Organs from Healthy Mice

**Figure 2:**
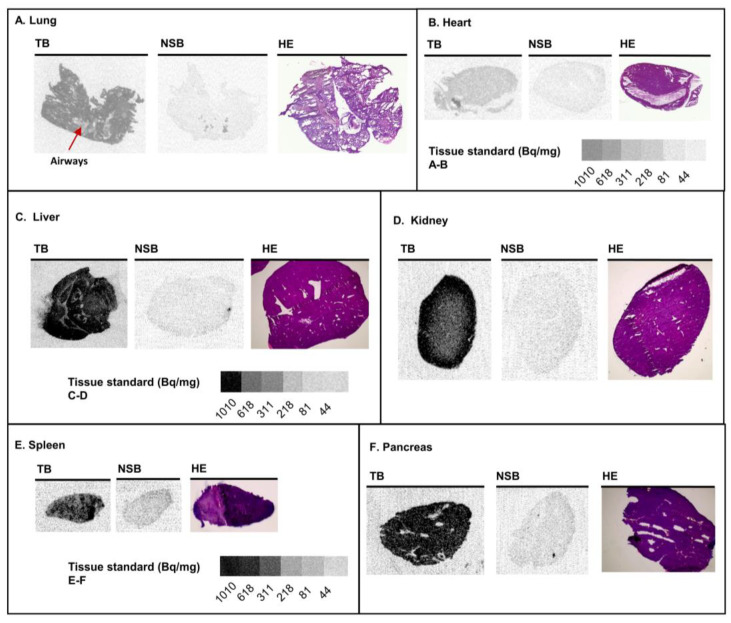
Representative images of tissue sections obtained from healthy female CD1 mice showing total radioligand binding (TB), non-specific binding (NSB; determined in presence of 5 µM FR), and hematoxylin-eosin staining (HE) of (**A**) lung, (**B**) heart, (**C**) liver, (**D**) kidney, (**E**) spleen, and (**F**) pancreas. Organ slices were incubated with 10 nM of [³H]PSB-15900-FR for 1 h at room temperature. Tissue equivalent standards are displayed below the respective panels.

**Figure 3:**
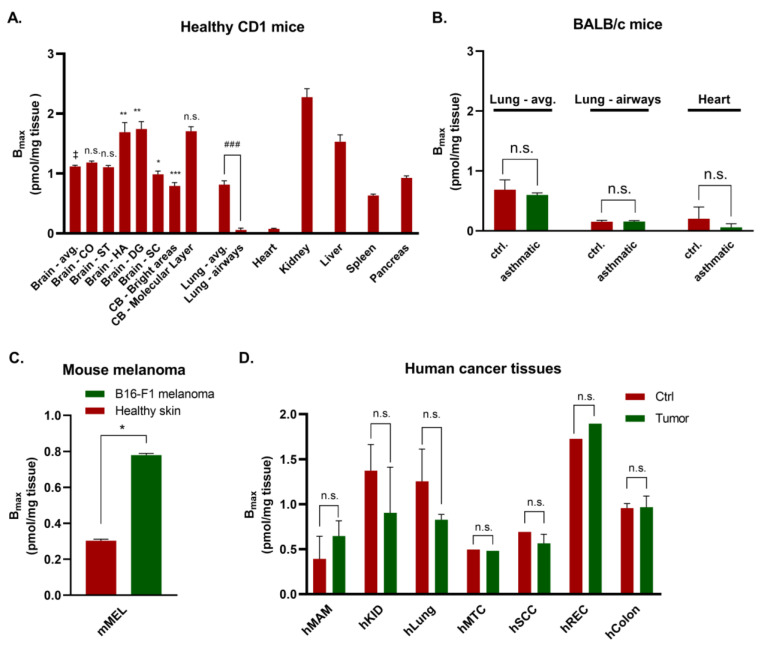
Gα_q/11_ protein expression (B_max_, given in pmol Gα_q/11_ proteins per mg tissue) of (**A**) tissues obtained from healthy female CD1 mice, (**B**) BALB/c mice treated with ovalbumin to induce acute asthma (asthmatic) or untreated BALB/c mice (ctrl.), (**C**) mice with B16-F1 melanoma and healthy skin as control, (**D**) samples from human cancers and surrounding healthy tissue. Values are presented as mean ± SEM from three (**A**) or two (**B**) individual animals, each measured at least in triplicate. avg., average; CO, cortex; ST, striatum; HA, hippocampus; DG, dentate gyrus; CB, cerebellum; mMEL, mouse melanoma; hMAM, human mammary gland; hKID, human kidney; hLung, human lung; hHNC, human head and neck; hSCC, human squamous cell carcinoma; hREC, human rectum; hColon, human colon. Comparisons between two mean values were performed by an unpaired, two-sided *t*-test, if necessary, corrected for multiple comparisons; if more than two mean values were compared, a one-way ANOVA with Dunnett’s post-hoc test was employed, in which all means were compared to the tissue average (mouse brain). Significance levels are defined as follows: n.s., *p* > 0.05; *, *p* < 0.05; **, *p* < 0.01, ***, *p* < 0.001, the reference value is indicated with ‡ in case of multiple comparisons. The significance level of the direct comparison between lung average and airways is denoted with # instead of *.

Expression levels and distribution of Gα_q_ proteins were further determined in tissue slices of the lung, heart, liver, kidney, spleen, and pancreas using [³H]PSB-15900-FR (see Figure 2 for images of total radioligand binding, nonspecific binding, and HE-staining, and Figure 3 and Table 1 for calculated expression levels of Gα_q_ proteins in all of the investigated tissues). In lung tissue, [³H]PSB-15900-FR displayed lower specific binding, as in the brain, with an average expression level of 0.81 pmol Gα_q_ proteins per mg tissue. In lung sections, radioligand was pronounced in the parenchyma, yielding higher values in whole lung sections when compared to the region of airways, accompanying blood vessels, and their surrounding tissue (arrow indicates as “airways”, see Figure 2A). In hearts, we observed only low, homogeneous expression of Gα_q_ proteins in the muscle tissue (0.08 pmol Gα_q_/mg tissue) with occasional dark spots, corresponding to blood clots in the ventricles (Figure 2B).

Liver tissue sections displayed a high average expression of Gα_q/11_ proteins (1.53 pmol Gα_q_/mg tissue), which was mostly homogenous, but contained structures displaying lower Gα_q/11_ protein expression (Figure 2C). These may represent bile ducts, vessels, or artifacts from cryosectioning (see HE-staining in Figure 2C). Kidney sections displayed very high Gα_q_ protein expression (2.27 pmol Gα_q_/mg tissue), especially in the renal cortex, but lower Gα_q_ protein expression in the medulla, which correlates with the lower cell density in the collecting duct system in the center of the kidney (Figure 2D). Spleen sections showed rather homogeneous [³H]PSB-15900-FR binding of intermediate expression levels (Figure 2E, 0.63 pmol Gα_q_/mg tissue). In sections of the pancreas, intermediate levels of Gα_q_ protein expression were observed (0.93 pmol Gα_q_/mg tissue). Areas displaying no specific binding included excretory ducts and septa, which subdivide the pancreas into irregular lobes (see Figure 2F).

### 3.3. Mouse Disease Models

**Figure 4:**
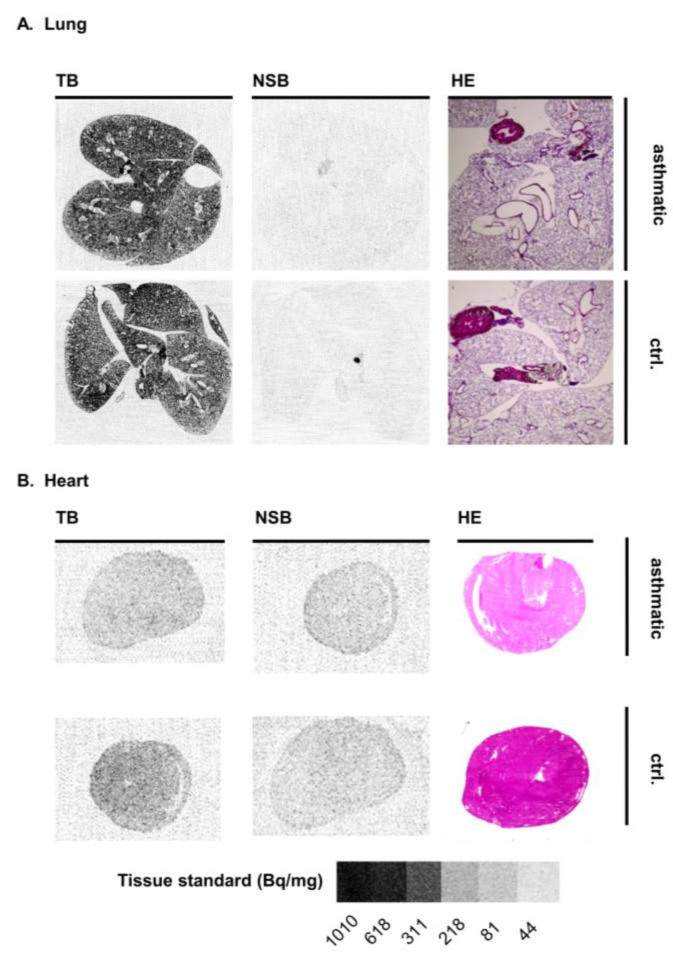
Representative images of (**A**) lung and (**B**) heart tissue sections obtained from BALB/c mice, which were either exposed to ovalbumin to induce symptoms of acute asthma (asthmatic) or were left untreated (ctrl.). TB, total radioligand binding; NSB, non-specific binding (determined in the presence of 5 µM FR), HE, hematoxylin-eosin staining. For autoradiography, organ slices were incubated with 10 nM [³H]PSB-15900-FR for 1 h at room temperature. Tissue equivalent standards are displayed at the bottom.

In a previous study, inhalation of FR had prevented bronchoconstriction in mouse models of ovalbumin-induced acute asthma bronchiale [18]. To investigate whether Gα_q_ protein expression levels are increased under these disease conditions, we imaged the Gα_q_ distribution in lung and heart sections of BALB/c mice using the same asthma model and compared the images to those of untreated BALB/c mice (Figure 4 and Figure 3B). The lungs were filled with modified OCT compound via the trachea before generating cryo-sections to prevent a collapse of the alveoli during sectioning. Similar to the observations in healthy lung tissue, the radioligand bound mainly to the lung parenchyma, while airways, blood vessels, and their surrounding tissue exhibited lower radioligand binding. No clear difference between the lungs of asthmatic mice and healthy mice could be observed (Figure 3B and Figure 4A; 0.60 and 0.69 pmol Gα_q_/mg tissue in asthmatic and healthy lungs, respectively). Furthermore, HE-stained lung tissues did not show any notable differences in the lung tissues between asthmatic and control mice (Figure 4A). Heart sections of mice with acute asthma displayed low, homogeneous levels of radioligand binding, similar to those of control mice (Figure 3B and Figure 4B; 0.06 and 0.21 pmol Gα_q_/mg tissue, respectively).

**Figure 5:**
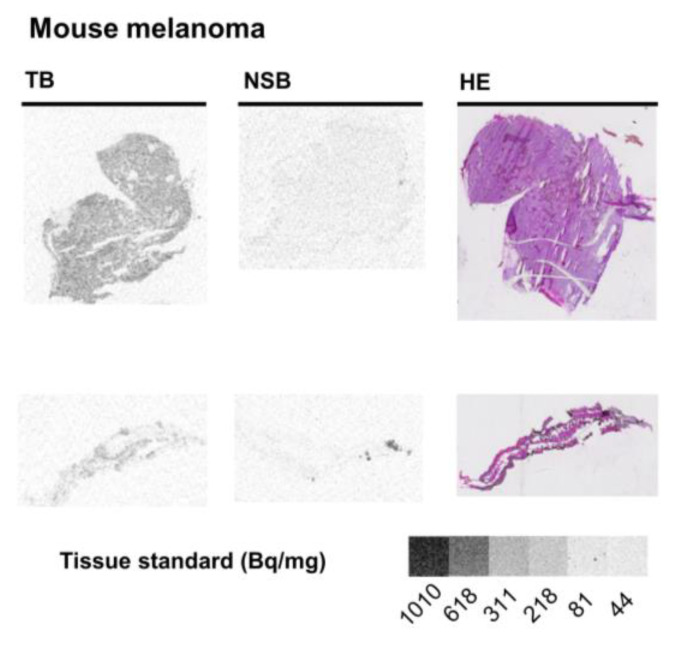
Representative images of sections from mouse melanoma and healthy skin. TB, total radioligand binding; NSB, non-specific binding (determined in the presence of 5 µM FR), HE, hematoxylin-eosin staining. For autoradiography, organ slices were incubated with 10 nM [³H]PSB-15900-FR for 1 h at room temperature. Tissue equivalent standards are displayed at the bottom.

Subsequently, we analyzed the Gα_q_ protein expression in a mouse cutaneous B16-F1 melanoma model. Melanoma tissue sections bound significantly more radioligand than healthy mouse skin (ca. 2.5-fold; see Figure 3C and Table 1; 0.78 and 0.30 pmol Gα_q_/mg tissue, respectively). Moreover, it displayed a morphology notably different from that of healthy skin samples (Figure 5).

### 3.4. Human Tissues

Next, we analyzed the expression and the distribution of Gα_q_ proteins in human tissues, namely in tumor samples and adjacent healthy tissues (see Figure 3D and Table 1 for expression levels).

**Figure 6:**
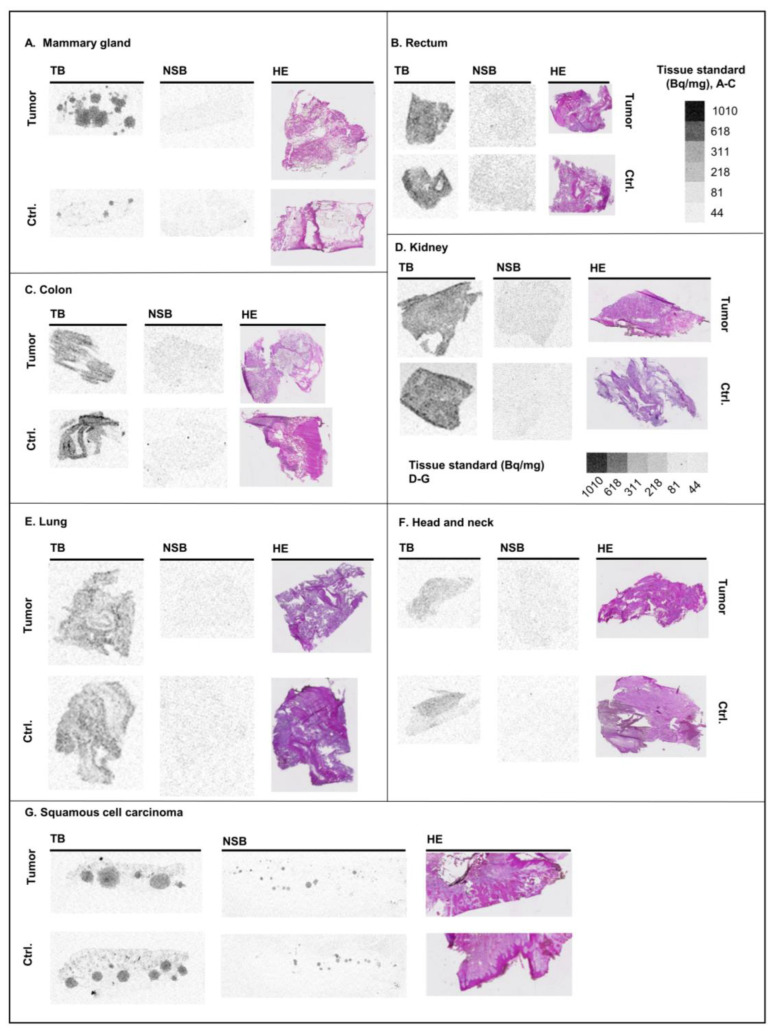
Representative images of cancer sections and healthy adjacent tissue obtained from the same individual. (**A**) mammary gland, (**B**), rectum, (**C**) colon, (**D**) kidney (**E**) lung, (**F**) head and neck, (**G**) squamous cell carcinoma. TB, total radioligand binding; NSB, non-specific binding (determined in the presence of 5 µM FR), HE, hematoxylin-eosin staining. For autoradiography, organ slices were incubated with 10 nM [³H]PSB-15900-FR for 1 h at room temperature. Tissue equivalent standards are displayed at the bottom right.

In mammary glands, both healthy and cancer tissue displayed low Gα_q_ protein expression (0.39 and 0.65 pmol Gα_q_/mg tissue, respectively). The expression appeared to be higher in the breast cancer as compared to the healthy tissue, but the difference was not significant (*p* = 0.45). All slices contained roughly circular spots of high radioligand binding, which appeared to be slightly larger in carcinoma samples and might correspond to drops of fat in the tissues, where the lipophilic radiotracer may have accumulated (Figure 6A). Rectum samples displayed high Gα_q_ protein expression, which was homogeneous in all cases and not different between control and tumor tissue (Figure 6B; 1.73 and 1.89 pmol Gα_q_/mg tissue respectively). Colon samples showed intermediate levels of Gα_q_ proteins and included areas of lower radioligand binding that could not be clearly matched to any specific structure when compared to HE-stained samples (Figure 6C; 0.96 and 0.97 pmol Gα_q_/mg tissue for control and tumor samples, respectively). Kidney samples displayed a rather homogeneous radioligand distribution of relatively high specific binding, however, one of the tumor cases bound almost no radioligand (Figure 6D; 1.37 and 0.83 pmol Gα_q_/mg tissue for control and tumor samples, respectively). The Gα_q_ expression in the human kidney was lower than in the mouse kidney. In lung samples, both healthy and tumor tissues bound similar amounts of radioligand (1.25 and 0.83 pmol Gα_q_/mg tissue for control and tumor samples, respectively), showing a non-homogenous radioligand distribution with no sharp contrasts as observed e.g., in mouse lung and brain (Figure 6E). Biopsies of the lung contained almost no lung parenchyma, but rather consisted of connective tissue and are therefore not comparable with the results that we had obtained from mouse lungs. Head and neck cancer tissues displayed a rather low radiotracer binding, similar to the surrounding healthy tissue (Figure 6F; 0.50 and 0.48 pmol Gα_q_/mg tissue for control and tumor samples, respectively). The samples were of small size and did not show histologically distinctive features. Squamous cell skin carcinoma and native skin displayed low to medium radioligand binding containing several large dark spots, similar to the mammary gland samples, where the radiotracer may have accumulated in droplets of subcutaneous fat (Figure 6G; 0.69 and 0.59 pmol Gα_q_/mg tissue for control and tumor samples, respectively). Radioligand binding was slightly higher in the epidermis than in the dermis. Compared to the B16-F1 melanoma, cancer originating from squamous cells did not show significantly increased Gα_q_ protein expression relative to healthy skin.

None of the human cancer samples displayed significantly altered Gα_q_ protein expression compared to adjacent healthy tissue (Figure 3D). Data from human tissues have to be interpreted with caution due to the quality of the samples. In addition, biopsy material may not be mainly composed of the characteristic tissue of the respective organ (e.g., lung parenchyma in lung samples), while tumor samples may be only partly infiltrated by malignant tissue and its stroma.

## 4. Discussion

The aim of the present study was to image and quantify the expression levels of Gα_q_ proteins in diverse tissues in health and disease. We hypothesized that certain pathological conditions might lead to an up- or downregulation of Gα_q_ protein expression in the affected organs or tissues. Autoradiography is a particularly well-suited method for quantifying protein expression, superior to other methods. In contrast to mRNA analysis, autoradiography directly measures protein expression, which in many cases does not correlate well with mRNA expression [25]. Furthermore, mRNA expression analysis in organ sub-structures is challenging due to difficulties in separating different cell types. Protein expression analysis by Western blotting cannot resolve protein distribution within a sample and yields only semi-quantitative results. Similarly, immunohistochemical staining does not provide quantitative protein expression levels, and may additionally be confounded by the reactivity of antibodies towards proteins with similar protein sequences. Moreover, antibodies cannot penetrate the cell membranes of intact cells to label the intracellular Gα_q_ proteins.

Both radioligands bind exclusively to the Gα_q_, Gα_11_, and Gα_14_ proteins, and not to the Gα_15_ protein at low nanomolar concentrations used in this study. Gα_15_ protein expression is, however, mostly tied to the hematopoietic system, e.g., blood and immune cells [12,13], and therefore all organs investigated in this study, with exception of the spleen, are not expected to contain relevant amounts of Gα_15_ proteins. Notably, the sequence of Gα_q_ family proteins is highly conserved across mammalian species, and the radiotracers display a virtually identical affinity to mouse and human Gα_q_ proteins [21]. This allowed us to investigate the expression levels and distribution of Gα_q_ family proteins in mice and humans using the same tracers, and, beyond that, facilitate their use in further pre-clinical contexts.

Radiotracers for autoradiography require a high binding affinity, and preferably a long residence time at the target protein, accompanied by low off-target and non-specific binding. These requirements are met by both investigated radioligands, as demonstrated in initial experiments showing that both radioligands, [³H]PSB-15900-FR and [³H]PSB-16254-YM, displayed high affinity binding to mouse brain, and nearly no non-specific binding after addition of high concentrations (5 µM) of FR. Both radioligands showed identical binding patterns in the brain, allowing a distinction between adjacent brain regions that expressed different levels of Gα_q/11_ proteins. Gαq protein expression was higher in the nerve fibers than in the cell soma, as indicated by a comparison between total radioligand binding and Nissl staining (Figure 1). This was unexpected since receptors (and therefore also G proteins) are expected to be expressed in dendrites, where they facilitate communication between nerve cells.

The quantification of Gα_q_ protein expression in brain sections resulted in virtually identical calculated expression levels for both radioligands (Figure 1D). In addition, both radioligands displayed similar affinity values and association rates, but [³H]PSB-15900-FR exhibited a dramatically increased residence time at the Gα_q_ protein, and therefore we continued our investigations by exclusively using the FR-derived radiotracer in this study.

The native expression of Gα_q_ proteins in the brain is ≈ 7-fold higher when compared to the typical expression levels of specific GPCRs in native tissue, e.g., a recent autoradiography-based study reported expression levels of approx. 300 fmol/mg tissue for the adenosine A_2A_ receptor in mice striatum (where the receptor is highly expressed) [26]. This makes sense since approx. 800 different GPCRs signal via only 16 Gα proteins, out of which three (Gα_q_, Gα_11_, and Gα_14_) were labeled by the radiotracer used in this study. About 45% of the therapeutically relevant receptors can signal via Gα_q_ family proteins [9,11]. High expression levels of Gα_q_ proteins in kidneys and livers point to a major role of Gα_q_-coupled receptor signaling in these organs. A role of purinergic GPCR signaling in kidney disease was previously proposed, and several P2Y receptors, such as the P2Y_2_ receptor, are expressed in the kidney and coupled to Gα_q_ proteins [27,28]. In the liver, GPCR-mediated signaling of metabolites (such as amino acids, lipids, and small carboxylic acids) may importantly contribute to Gα_q_ signaling [29].

In this study, we detected very low Gα_q_ protein expression levels in the mouse heart, which may, however, have been increased in embryonic development [15,30]. Gα_q_ overexpression in the adult heart has been associated with cardiac hypertrophy [20].

As Gα_q_ proteins are involved in signal transduction in *asthma bronchiale* [18], we compared the Gα_q_ protein expression in the lung and additionally in heart sections of BALB/c mice with acute asthma to that of untreated mice. No significant difference of Gα_q_ protein expression was found between healthy and asthmatic mice, neither in the lung tissue, the airways, nor the heart. This indicates that acute asthma does not induce an increase in Gα_q_ protein expression, and airway constriction is rather caused by hyperactivation of Gα_q_ proteins and not by overexpression. However, in chronic asthma associated with airway remodeling [31], the expression levels of disease-associated proteins, such as the Gα_q_ protein, may change. Expression levels of Gα_q_ proteins were very similar in lung and heart tissue sections of different mouse strains (CD1 and BALB/c), which also confirms that the determination of Gα_q_ protein expression levels by autoradiography using [³H]PSB-15900-FR is highly reproducible.

Mouse melanoma exhibited a significantly higher expression of Gα_q_ proteins than healthy skin (see Figure 5). Thus, Gα_q_ proteins and their increased signaling may not only contribute to the malignancy of uveal melanoma, which typically shows specific mutations in Gα_q_ proteins leading to their hyperactivation, but also to that of cutaneous melanoma not harboring mutations in the Gα_q_ protein [17,32,33]. Due to the increased expression of Gα_q_ proteins in melanoma, diagnostics based on an FR-scaffold may be useful to identify melanoma cells and to locate metastases in vivo. Furthermore, Gα_q_ protein inhibitors could be suitable as therapeutics, in view of the toxicity at therapeutic concentrations, especially for local therapy [34].

In the investigated human cancer samples (which did not include melanoma), we could not detect significant differences in Gα_q_ protein expression compared to healthy tissue from the same patients. All human samples used in this study were obtained from a Biobank and were not freshly resected from patients. Image quality, however, is best with freshly obtained, snap-frozen material. Compared to mouse samples, sections of human material displayed considerably more artifacts, e.g., high variability in radioligand binding without corresponding histological changes within a single section, or fissures in the tissue. Additionally, biopsies were mostly taken from different sites within an organ, which hinders direct comparison. Nevertheless, due to large differences in expression levels in different organs and tissues, the Gα_q_ tracer might be useful to localize metastases, even when the Gα_q_ expression level is not increased in the cancer cells.

In summary, this study provides a comprehensive analysis of the expression and distribution of Gα_q_ proteins in several vital organ systems of healthy mice, disease models, and samples of human cancers and adjacent healthy tissues. We found that ³H-labeled radiotracers derived from macrocyclic Gα_q_ protein inhibitors bound with high selectivity and allowed quantitative high-resolution measurements of Gα_q_ protein expression. While we have not detected any up-or-downregulation of Gα_q_ proteins in a mouse model of acute asthma, a significant increase in Gα_q_ protein expression was observed in a B16-F1 mouse melanoma model compared to healthy mouse skin samples. Due to the high affinity of both radiotracers, the hardly detectable non-specific binding, and their ability to discriminate between organ sub-structures, the future development of Gα_q_-protein binding radiotracers as novel diagnostics is highly promising.

## Figures and Tables

**Table 1:** B_max_ values (pmol of Gα_q/11_ proteins per mg tissue) obtained from autoradiography experiments in different mouse organs.

Mouse Tissues		Human Tissues	
Healthy Mouse Organs	B_max_ ± SEM (pmol/mg of Tissue)	Cancer Samples	B_max_ ± SEM (pmol/mg of Tissue)
Brain—average	1.12 ± 0.02	Mammary gland—control	0.39 ± 0.25
Brain—striatum	1.11 ± 0.03	Mammary gland—tumor	0.65 ± 0.17
Brain—cortex	1.18 ± 0.02	Kidney—control	1.37 ± 0.29
Brain—hip. sulc.	1.69 ± 0.16	Kidney—tumor	0.90 ± 0.51
Brain—gyr. dent.	1.74 ± 0.12	Lung—control	1.25 ± 0.36
Brain— superior colliculus	0.99 ± 0.06	Lung—tumor	0.83 ± 0.06
Cerebellum— Bright areas	0.79 ± 0.06	Head and neck—control	0.50
Cerebellum— Molecular layer	1.71 ± 0.07	Head and neck—tumor	0.48
Lung—average	0.81 ± 0.07	Skin—control	0.69
Lung—airways	0.06 ± 0.03	Skin—squamous cell carcinoma	0.56 ± 0.10
Heart	0.08 ± 0.01	Rectum—control	1.73
Kidney	2.27 ± 0.14	Rectum—tumor	1.89
Liver	1.53 ± 0.12	Colon—control	0.96 ± 0.05
Spleen	0.63 ± 0.02	Colon—tumor	0.97 ± 0.12
Pancreas	0.93 ± 0.04		
**Mouse disease models**			
Lung control—full	0.69 ± 0.17		
Lung asthmatic—full	0.60 ± 0.04		
Lung control—airways	0.15 ± 0.02		
Lung asthmatic—airways	0.16 ± 0.02		
Heart—control	0.21 ± 0.19		
Heart—asthmatic	0.06 ± 0.06		
Mouse—skin	0.30 ± 0.01		
Mouse—melanoma	0.78 ± 0.01		

## Data Availability

The data presented in this study are available on request from the corresponding author.
